# Neuroinflammatory changes in acute myeloid leukemia: Evidence for blood–brain barrier disruption and glial activation

**DOI:** 10.1002/hem3.70341

**Published:** 2026-03-30

**Authors:** Marta Febo, Daniele Sorcini, Alessandra Mirarchi, Paolo Cogliati, Arianna Stella, Paolo Sportoletti, Olga Ermakova, Martina Mandarano, Maria Cristina Marchetti, Carlo Riccardi, Brunangelo Falini, Graziella Migliorati, Cataldo Arcuri, Stefano Bruscoli, Oxana Bereshchenko

**Affiliations:** ^1^ Department of Medicine and Surgery University of Perugia Perugia Italy; ^2^ Italian National Research Council Institute of Biochemistry and Cell Biology IBBC Rome Italy; ^3^ Department of Philosophy, Social Sciences, Humanities and Education University of Perugia Perugia Italy

Acute myeloid leukemia (AML) is the most aggressive form of hematologic malignancies in adults, driven by somatically acquired mutations in genes regulating myeloid lineage differentiation, proliferation, and survival.^1^ Among cytogenetically normal AML (CN‐AML), which accounts for 40%–50% of cases, recurrent mutations in *NPM1*, *FLT3*, and *DNMT3A* are most common.^2^ Mutations in NPM1 nuclear localization signal disrupt its nuclear function, but on their own are usually insufficient to induce AML, and cooperating lesions such as FLT3‐Internal tandem duplication (ITD) mutations are often required to promote leukemic transformation.^3^ FLT3‐ITD mutations lead to constitutive kinase activation, resulting in increased blast survival and more aggressive disease progression.^1^, ^3^

Several hematologic malignancies show persistent inflammation arising from both leukemia‐intrinsic and microenvironmental sources.^4^ Importantly, several AML‐associated mutations have been linked not only to leukemogenesis but also to dysregulation of inflammatory pathways. Somatic mutations can activate innate immune pathways, such as TLR–NF‐κB signaling, inflammasome activation, and type‐I interferon responses, within malignant cells, while altered stromal and immune cells in the bone marrow further amplify these inflammatory circuits. Elevated plasma levels of proinflammatory cytokines such as IL‐6, IL‐1β, TNF‐α, and IFN‐γ, which are commonly observed in AML patients, contribute to leukemic survival and resistance to therapies.^4^, ^5^ Epidemiologic data show increased AML incidence in individuals with autoimmune conditions,^6^, ^7^ indicating that pre‐existing inflammation may promote leukemogenesis. On the other hand, AML itself can act as a driver of systemic inflammation.

While these findings highlight the importance of inflammation in AML, it remains unclear whether AML‐associated inflammatory signals extend beyond the hematopoietic system to affect other organs, such as the central nervous system (CNS). This question is particularly relevant considering recent work showing that clonal hematopoiesis of indeterminate potential (CHIP) can drive systemic inflammation and influence neurological outcomes. For example, *TET2*‐driven CHIP lowers Alzheimer's disease (AD) risk,^8^ and brain‐infiltrating Tet2 mutant monocytic cells were protective in a mouse model of AD.^9^ In contrast, *DNMT3A*‐mutant monocyte‐derived microglia accumulate in nigrostriatal regions and induce Parkinson's disease‐like motor deficits,^10^ demonstrating that premalignant hematopoietic clones can modulate neuroinflammatory processes and neurological function. In addition, hematologic therapies, such as involving CAR‐T cells, can induce CNS inflammation and associated neurotoxicity, which arises from excessive cytokine release with endothelial activation and blood–brain barrier (BBB) disruption.^11^ Bleeding and coagulation abnormalities common in AML can also promote endothelial activation and BBB alterations, thereby contributing to inflammatory responses in the CNS. Together, these observations underscore the vulnerability of the CNS to inflammatory stimuli originating from hematopoietic cells or their therapies. Yet, whether inflammation driven by AML cells themselves contributes to CNS dysfunction has not been systematically investigated.

This question is particularly relevant, given the emerging role of inflammation in neuropsychiatric and neurological disorders.^12^ Elevated levels of cytokines such as IL‐1β, IL‐6, and TNF‐α have been associated with major depressive disorder and anxiety, and are thought to influence CNS function by altering neurotransmitter metabolism, synaptic plasticity, and behavior.^13^ Psychiatric disorders such as anxiety and depression affect approximately 20%–30% of patients with AML, a rate lower than that in patients with high‐prevalence solid tumors but higher than that in most chronic diseases.^14^ Although the psychological impact of a life‐threatening diagnosis is a clear risk factor for these conditions, it is still unknown whether the inflammatory milieu accompanying AML contributes biologically to these symptoms or to broader CNS dysfunction.

To investigate this possibility, we used a murine model of *NPMc⁺/Flt3‐ITD* AML, which rapidly develops leukemia and closely recapitulates human disease.^15^ Because previous studies have shown that altered NPM1 function can trigger aberrant inflammasome activation, while FLT3‐ITD promotes cytokine network alterations and a proinflammatory bone‐marrow microenvironment,^16^, ^17^ we hypothesized that AML‐related inflammation caused by these leukemogenic mutations may also induce neuroinflammation. Therefore, we evaluated CNS inflammatory responses in this genetically defined model of AML.

To this end, we first purified and quantified leukocytes from whole‐brain homogenates of leukemic mice defined as such by elevated peripheral white blood cell (WBC) counts and reduced hemoglobin levels compared to age‐matched healthy controls (Supporting Information S1: Tables 1 and 2). A significant increase in the total leukocyte count was detected in the brains of leukemic mice compared to controls (Figure 1A). Flow cytometric analysis revealed significantly increased numbers of CD4⁺ and CD8⁺ T cells, CD11b⁺CD45^hi^ myeloid cells, and CD11b⁺CD45^lo^ microglia in leukemic mice (Figure 1B,C). As normal monocytic differentiation is largely impaired in the *NPMc⁺/Flt3‐ITD* model, the expanded CD11b⁺CD45^hi^ population represents leukemic myeloid cells rather than bona fide monocytes. Consistently, CD11b⁺CD45^hi^ cells from leukemic brains showed increased forward scatter, consistent with enlarged cell size typical of leukemic cells (Figure 1D), and expressed Mac‐1 and Gr‐1, but not the markers of leukemia stem cells (Supporting Information S1: Figure S1A–J). The presence of CNS‐infiltrating leukemic blasts was confirmed by detection of the Npm1c transgene expression by qPCR (Figure 1E) and by immunofluorescence staining in spleen and brain parenchyma in *NPMc⁺/Flt3‐ITD* but not in control animals (Figure 1F,G). A significant positive correlation between peripheral WBC counts and CNS infiltration was observed (Spearman test, P = 0.018) (Supporting Information S1: Figure S1K). These results suggest that leukemic progression is associated with increased immune and leukemic cell infiltration into the brain. Consistent with a systemic inflammatory state, mRNA expression of the proinflammatory cytokines *Il1b*, *Il6*, and *Tnfa* was increased in spleens from *NPMc⁺/Flt3‐ITD* mice compared with controls (Figure 1H). The frequency of IL‐6–producing CD11b⁺ cells was also increased in peripheral blood of NPMc+/Flt3‐ITD as compared to control mice (Figure 1I).

**Figure 1:**
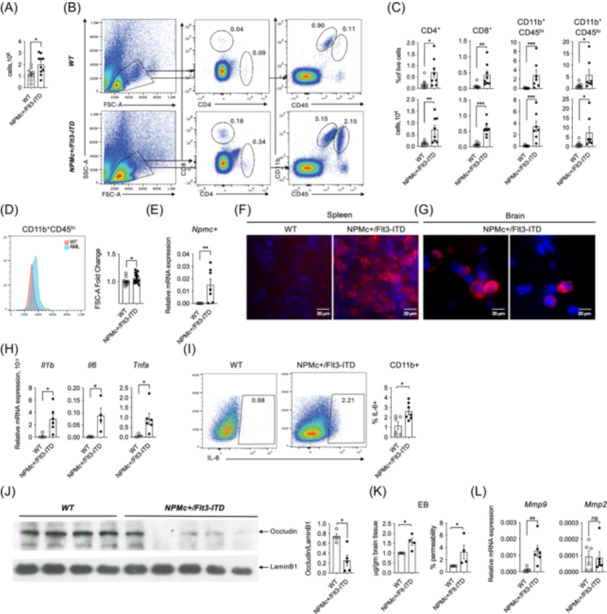
**Analysis of immune cell brain infiltration and blood–brain barrier integrity in** ***WT*** **and** ***NPMc⁺/Flt3‐ITD*** **mice**. **(A)** Total counts of leukocytes isolated from the brains of 3–8‐month‐old *WT* and *NPMc⁺/Flt3‐ITD* mice. **(B)** Flow cytometry analysis of brain immune cell populations from *WT* and *NPMc⁺/Flt3‐ITD* mice. The gating strategy includes CD8⁺CD4⁻ and CD4⁺CD8⁻ T cells, CD11b⁺CD45^hi^ monocytes/macrophages/leukemic cells, and CD11b⁺CD45^lo^ microglia. Numbers within quadrants indicate frequency within the parent population. **(C)** Quantification of immune cell subsets in the brain. Top panels show frequencies (% of total cells) and bottom panels show absolute numbers (×10^4^) of indicated cell types. **(D)** Flow cytometric analysis of the cell size (FSC‐A) of CD11b⁺CD45^hi^ cells isolated from the brains of *WT* and *NPMc⁺/Flt3‐ITD* mice. A representative histogram overlay is shown; the graph depicts the mean FSC‐A fold change. **(E)** Relative mRNA expression levels of *Npmc+* in brain tissue from 3 to 8‐month‐old *WT* and *NPMc⁺/Flt3‐ITD* mice assessed by qPCR. Data are presented relative to *Actb* expression. **(F)** Representative immunofluorescence images of spleen sections from *WT* and *NPMc⁺/Flt3‐ITD* mice stained for DAPI (blue, nuclei) and human NPM1 (red). Scale bars: 20 μm. **(G)** Representative immunofluorescence images of brain sections from *NPMc⁺/Flt3‐ITD* mice stained for DAPI (blue, nuclei) and human NPM1 (red). Scale bars: 20 μm. **(H)** Relative mRNA expression levels of proinflammatory cytokines in spleens from *W*T and *NPMc⁺/Flt3‐ITD* mice assessed by qPCR. All data are presented relative to *Actb* expression. **(I)** Flow cytometry analysis of intracellular IL‐6 expression in CD11b^+^ peripheral blood leukocytes from *WT* and *NPMc⁺/Flt3‐ITD* mice. Numbers within quadrants indicate frequency within the parent population. **(J)** Western blot analysis of occludin expression in brain lysates from *WT* and *NPMc*⁺*/Flt3‐ITD* mice. LaminB1 is used as a loading control. Quantification of occludin relative to LaminB1 is shown on the right. **(K)** Quantification of Evans Blue (EB) concentration (μg/gm of brain tissue, left panel) and brain vascular permeability in *WT* and *NPMc⁺/Flt3‐ITD* mice (right panel) following systemic dye injection. **(L)** Relative mRNA expression levels of *Mmp9* and *Mmp2* in brain tissue assessed by qPCR.  All data are presented relative to *Actb* mRNA expression. Each dot represents an individual mouse; bars indicate the mean. Results are presented as the means ± SEM of six **(D)**, five **(A**–**C)**, four **(K)**, three **(E**, **F**, **G**, **H**, **L)**, and two **(I)**, and representative of two **(J)** independent experiments (***P < 0.001, **P < 0.01, *P < 0.05, and ns, non significant, Mann–Whitney test). mRNA, messenger RNA.

The blood–brain barrier (BBB) plays a crucial role in maintaining CNS homeostasis by regulating immune cell trafficking.^18^ Tight junctions (TJs), primarily composed of occludin and claudin‐5, are essential for preserving BBB integrity by controlling intercellular permeability. Degradation of the TJ components, often mediated by matrix metalloproteinases (MMPs), compromises the BBB, increasing permeability and leukocyte access, thereby contributing to neuroinflammation and neurological disorders. Infections and inflammatory states are known to challenge BBB integrity.^18^

To assess BBB integrity in the brain of WT and leukemic mice, we evaluated the expression of the TJ component occludin via western blot analysis. Notably, occludin protein levels were significantly reduced in the brains of *NPMc⁺/Flt3‐ITD* mice compared to controls (Figure 1J and Supporting Information S1: Figure S2A–C). We next performed functional Evans Blue (EB) permeability assays to validate BBB disruption. Both EB accumulation in the brain and BBB permeability (brain‐to‐blood EB concentration ratio), expressed as fold change relative to WT controls, were significantly increased in AML mice (Figure 1K). Circulating EB levels did not differ significantly between groups (Supporting Information S1: Figure S2D,E), indicating that the elevated brain EB is not due to differences in systemic dye availability but reflects a functional decrease in BBB integrity. To determine whether dysregulated MMP expression may contribute to TJ degradation, we measured mRNA levels of *Mmp2* and *Mmp9* by quantitative real‐time PCR (qPCR). Our results showed a significant increase in *Mmp9* expression levels in *NPMc⁺/Flt3‐ITD* mice compared to controls, while *Mmp2* levels did not differ significantly between the groups (Figure 1L). These findings suggest that MMP9‐mediated TJ degradation may contribute to BBB disruption and facilitate leukocyte infiltration into the brain.

To investigate whether the enhanced leukocyte infiltration contributes to the establishment of a neuroinflammatory microenvironment in the brains of the leukemic mice, we measured the mRNA expression levels of the expression of key inflammatory mediators in whole‐brain tissue from WT and leukemic mice. Specifically, we measured mRNA levels of pro‐ and anti‐inflammatory cytokines (*Il1b, Tnfa, Il6,* and *Tgfb*), chemokines involved in immune cell recruitment (*Ccl2* and *Ccl4*), homeostatic and neuroprotective chemokines (*Cx3cl1, Cxcl12,* and *Cxcl9*), and type I interferons (*Ifna* and *Ifnb*). Leukemia‐bearing mice showed significantly elevated expression of *Il1b, Tnfa, Il6, Tgfb, Ccl2*, and *Ccl4*, while *Cx3cl1, Cxcl12*, and *Cxcl9* were markedly downregulated; however, expression of *Ifna* and *Ifnb* remained unchanged (Figure 2A). These data suggest a brain‐restricted inflammatory response marked by immune cell recruitment, glial activation, and loss of homeostatic signals, rather than systemic immune activation. Collectively, these results indicate a selective upregulation of proinflammatory cytokines in the brains of leukemic mice, consistent with a brain‐associated innate inflammatory response.

**Figure 2:**
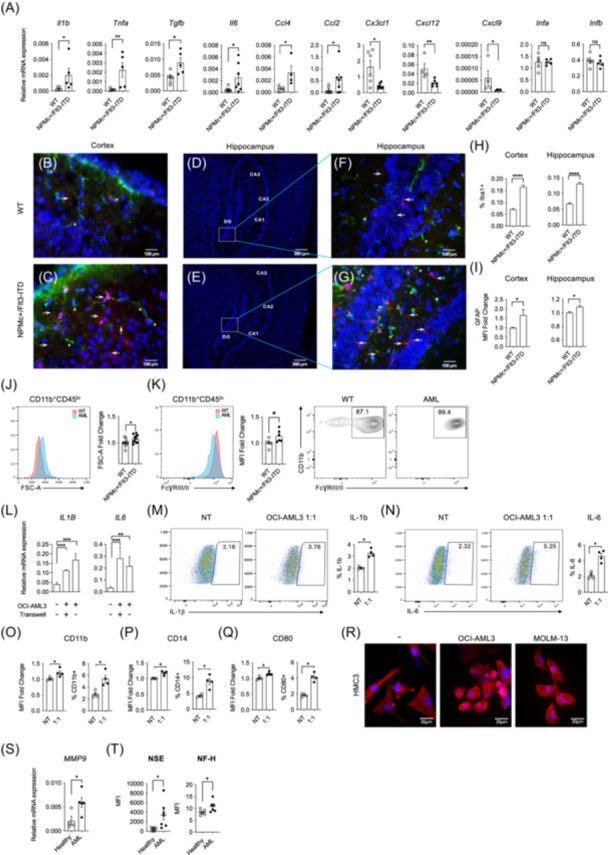
**AML induces neuroinflammatory responses in vivo and in vitro**. **(A)** Relative mRNA expression levels of pro‐ and anti‐inflammatory cytokines in brain tissue from 3 to 8‐month‐old *WT* and *NPMc*⁺*/Flt3‐ITD* mice assessed by qPCR. Data are presented relative to *Actb* expression. **(B**, **C)** Representative immunofluorescence images of the cortex from *WT* **(B)** (*n* = 6) and *NPMc*⁺*/Flt3‐ITD* **(C)** mice (*n* = 7). Sections were stained for Iba1 (red, microglia), GFAP (green, astroglia), and DAPI (blue, nuclei). Arrows indicate Iba1‐positive microglia and asterisks indicate GFAP‐positive astroglia. Scale bars: 100 μm. **(D**, **E)** Overview images of the hippocampus, showing CA1, CA2, CA3, and dentate gyrus (DG) regions in WT **(D)** (*n* = 6) and *NPMc*⁺*/Flt3‐ITD* **(E)** (*n* = 7) mice stained with DAPI. Scale bars: 300 μm. **(F**, **G)** Higher magnification images of the DG region from *WT* **(F)** and *NPMc*⁺*/Flt3‐ITD* **(G)** mice. Scale bars: 100 μm. Arrows indicate Iba1‐positive microglia and asterisks indicate GFAP‐positive astroglia. **(H)** Quantification of Iba1⁺ microglial cells in the cortex (left panel) and the hippocampus (right panel) of *WT* and *NPMc⁺/Flt3‐ITD* mice. Quantification was performed on 10 representative images per genotype (*n* = 3). Results are expressed as percentage of Iba1⁺ cells relative to total nuclei. **(I)** Quantification of GFAP expression in the cortex (left panel) and the hippocampus (right panel) of *WT* and *NPMc⁺/Flt3‐ITD* mice. Graphs show the mean fluorescence intensity (MFI) fold change. **(J)** Flow cytometric analysis of cell size (FSC‐A) of microglia (CD11b⁺CD45^lo^) isolated from brains of *WT* and *NPMc⁺/Flt3‐ITD* mice. A representative histogram overlay is shown; the graph depicts the mean FSC‐A fold change. **(K)** Flow cytometry analysis of FcγRIII/II (CD16/32) expression in microglia (CD11b⁺CD45^lo^) from WT and NPMc⁺/Flt3‐ITD mice. A representative histogram overlay is shown; the graph displays the mean fluorescence intensity (MFI) fold change. The representative plot to the right shows the comparative frequency of FcγRIII/II⁺ microglia between groups. **(L)** Relative expression of *IL1B* and *IL6* mRNA in the HMC3 human microglia cell line co‐cultured with OCI‐AML3 cells, with or without direct cell‐to‐cell contact. **(M**, **N)** Flow cytometry analysis of intracellular IL‐1β **(M)** and IL‐6 **(N)** expression in HMC3 cells cultured under nontreated (NT) conditions or exposed to OCI‐AML3‐conditioned medium (1:1 in DMEM 10% FBS). Numbers within quadrants indicate the frequency of the gated population. **(O**–**Q)** Flow cytometry analysis of CD11b **(O)**, CD14 **(P)**, and CD80 **(Q)** expression in HMC3 cells cultured under NT conditions or exposed to OCI‐AML3‐conditioned medium. Graphs show the MFI fold change (left panels) and the percentage of gated populations (right panels). **(R)** Morphological analysis of HMC3 cells exposed for 24 h to OCI‐AML3‐ or MOLM‐13‐conditioned medium (1:2 in DMEM 10% FBS). Microglial cells were stained with phalloidin to detect F‐actin (red) and DAPI to visualize nuclei (blue). Morphological changes indicate activation and cytoskeletal remodeling. Scale bars: 20 μm. **(S)** Relative expression of *MMP9* mRNA in mononuclear cells isolated from peripheral blood of AML patients and healthy donors. **(T)** Quantification of neuronal damage markers NSE, NF‐H in plasma isolated from peripheral blood of AML patients and healthy controls, using the ProcartaPlex™ Human Brain Injury Panel (5‐Plex; Thermo Fisher Scientific) based on Luminex xMAP® technology. Each dot represents an individual mouse/patient; bars indicate the mean. Results are presented as the means ± SEM of six **(J)**, four **(B**–**I)**, three **(A**, **K**, **L)**, and two **(M**–**Q)**, and are representative of one **(R**–**T)** independent experiment (****P < 0.0001,***P < 0.001, **P < 0.01, *P < 0.05, and ns, non significant, Mann–Whitney test). DAPI, 4′,6‐diamidino‐2‐phenylindole; GFAP, glial fibrillary acidic protein; mRNA, messenger RNA; qPCR, quantitative polymerase chain reaction.

To determine whether glial activation accompanies this inflammatory state, we examined astroglial and microglial activation in the cortex and hippocampus of WT and leukemia‐bearing mice by immunofluorescence staining of brain sections with glial fibrillary acidic protein (GFAP), ionized calcium‐binding adaptor molecule 1 (Iba1), and DAPI to label astrocytes (green), microglia (red), and nuclei (blue), respectively, as we previously described.^19^ The number of GFAP‐positive astrocytes (asterisks) and Iba1‐positive microglia cells (arrows) was significantly increased in cortex sections of *NPMc*+*/Flt3‐ITD* mice compared to controls (Figure 2B,C and data not shown), suggesting reactive astrogliosis and microglia activation.

Similar glial alterations were observed in the hippocampus. Low‐magnification images show that the hippocampal architecture was preserved in both groups (Figure 2D,E), while high‐magnification images of the dentate gyrus (DG) revealed increased numbers of GFAP‐positive astrocytes and Iba1‐positive microglia in *NPMc⁺/Flt3‐ITD* mice compared to controls (Figure 2F,G and data not shown). The reactive status of astrocytes in leukemic mice was characterized by enhanced expression of GFAP and more ramified morphology (arrows). Objective quantification of images corroborated the histological findings, revealing a significant increase in the proportion of Iba1⁺ microglia cells (Figure 2H) and GFAP expression (Figure 2I) in both the cortex and the hippocampus of *NPMc⁺/Flt3‐ITD* mice compared to controls. To investigate the phenotypic features associated with microglial activation, we performed flow cytometric analysis of CD11b⁺CD45^lo^ microglia isolated from the brains of WT and *NPMc⁺/Flt3‐ITD* mice. Microglia from leukemic mice showed a significant increase in the forward scatter area (FSC‐A), suggesting a hypertrophic, activated state (Figure 2J). In addition, the expression of FcγRIII/II (CD16/32), a marker associated with proinflammatory microglial activation,^20^ was significantly upregulated in microglia from *NPMc⁺/Flt3‐ITD* mice compared to controls (Figure 2K). Taken together, these data suggest that leukemic mice show a glial phenotype indicative of ongoing neuroinflammation.

In addition, to explore whether human AML cells may directly lead to microglial activation, we used a co‐culture system using HMC3 human microglia and OCI‐AML3 AML cell lines (carrying *NPM1* mutation A and WT *FLT3*). The two cell lines were co‐cultured either in direct cell‐to‐cell contact or in an indirect trans‐well setting, allowing for the exchange of soluble factors without direct cell contact. Consistent with microglial activation, AML‐conditioned media induced IL‐1β and IL‐6 production (Figure 2L–N) and increased expression of CD11b, CD14, and CD80 in HMC3 cells (Figure 2O–Q, Supporting Information S1: Figure S3A–C), whereas CD86 expression remained unchanged (Supporting Information S1: Figure S3D). Moreover, morphological assessment of HMC3 microglia cells exposed to OCI‐AML3‐ or MOLM‐13 (*NPM1* WT and *FLT3‐ITD*‐positive)‐conditioned media showed marked morphological transformation characterized by a more rounded shape, consistent with the activated state (Figure 2R). Thus, the data obtained *in vitro* using human cell lines further suggest that AML cells may cause microglial activation via secretion of soluble factors.

To extend our in vitro observations to the clinical setting, we assessed whether *MMP9* expression is also increased in human AML patients. For this purpose, we compared the *MMP9* mRNA expression in peripheral blood mononuclear cells isolated from AML patients and healthy donors. Consistent with murine data, *MMP9* expression was significantly elevated in AML samples compared to controls (Figure 2S). Although differences in MMP9 protein levels or activity await confirmation, the upregulation of *MMP9* mRNA both in mice and in human AML suggests that it may play a role in BBB disruption. The observed upregulation of MMP9 transcription supports our hypothesis and strengthens the translational relevance of our murine findings.

Finally, to determine whether the AML‐associated inflammatory responses may contribute to CNS alterations in AML patients, we compared levels of known neuronal injury markers in plasma of AML patients and healthy controls using multiplex technology. We detected significantly elevated levels of neuron‐specific enolase (NSE) and neurofilament heavy chain (NF‐H) in plasma of AML patients compared to those of healthy controls (Figure 2T and Supporting Information S1: Figure S4A). Importantly, mRNA expression of these neuronal markers did not significantly differ in PBMC from AML patients and healthy controls (Supporting Information S1: Figure S4B). The increase in plasma levels of two independent protein markers of neuronal damage suggests the presence of a possible AML‐associated neuroinflammation in human AML.

Our findings provide evidence that AML progression is associated with neuroinflammatory changes characterized by BBB disruption, increased leukocyte infiltration, glial activation, and elevated levels of pro‐inflammatory cytokines. In addition, both in vitro data and patient‐derived evidence support the potential involvement of AML in the onset of neuroinflammation and its consequent neurological manifestations. Understanding how systemic malignancies like AML induce brain‐specific inflammatory responses may provide new avenues for neuroprotective strategies aimed at improving cognitive outcomes and the overall quality of life in leukemia patients. The selective cytokine profile points to specific inflammatory pathways that might be therapeutically targeted to mitigate CNS involvement in leukemia. Future studies should explore whether modulating neuroinflammation can alleviate these neurological effects and examine the long‐term impact of leukemia‐induced glial activation on brain structure and function.

## AUTHOR CONTRIBUTIONS

**Marta Febo**: Investigation; writing—original draft; writing—review and editing; data curation; methodology. **Daniele Sorcini**: Investigation; resources; methodology; writing—review and editing; data curation. **Alessandra Mirarchi**: Investigation; methodology. **Paolo Cogliati**: Investigation; methodology; validation. **Arianna Stella**: Investigation; writing—review and editing; methodology. **Paolo Sportoletti**: Writing—review and editing; methodology; resources. **Olga Ermakova**: Writing—review and editing; methodology; formal analysis. **Martina Mandarano**: Investigation; methodology. **Maria Cristina Marchetti**: Investigation; methodology; data curation. **Carlo Riccardi**: Writing—review and editing; formal analysis. **Brunangelo Falini**: Methodology; writing—review and editing; resources; project administration. **Graziella Migliorati**: Writing—review and editing; project administration; supervision. **Cataldo Arcuri**: Investigation; conceptualization; writing—review and editing; methodology; data curation; formal analysis. **Stefano Bruscoli**: Investigation; writing—review and editing; methodology; formal analysis; data curation; supervision; funding acquisition. **Oxana Bereshchenko**: Investigation; conceptualization; writing—original draft; writing—review and editing; validation; formal analysis; project administration; data curation; supervision; methodology.

## CONFLICT OF INTEREST STATEMENT

The authors declare no conflict of interest.

## FUNDING

This research was funded by the Italian Ministry of University and Research grant (PRIN‐202039WMFP) to S.B.

## Supporting information

Supporting Figures Rev2‐ 19022026.

Supplemental_Tables_Rev2.

## Data Availability

The data that support the findings of this study are available from the corresponding author upon reasonable request.
