# Supplementary material for: Neuroinflammatory changes in acute myeloid leukemia: Evidence for blood–brain barrier disruption and glial activation
Source: Hemasphere. 2026 Mar 30;10(4):e70341. doi: 10.1002/hem3.70341 (PMC13103871; doi:10.1002/hem3.70341)
Supplement: Supplementary file 1 — Supporting Figures Rev2‐ 19022026. [file HEM3-10-e70341-s001.pdf]

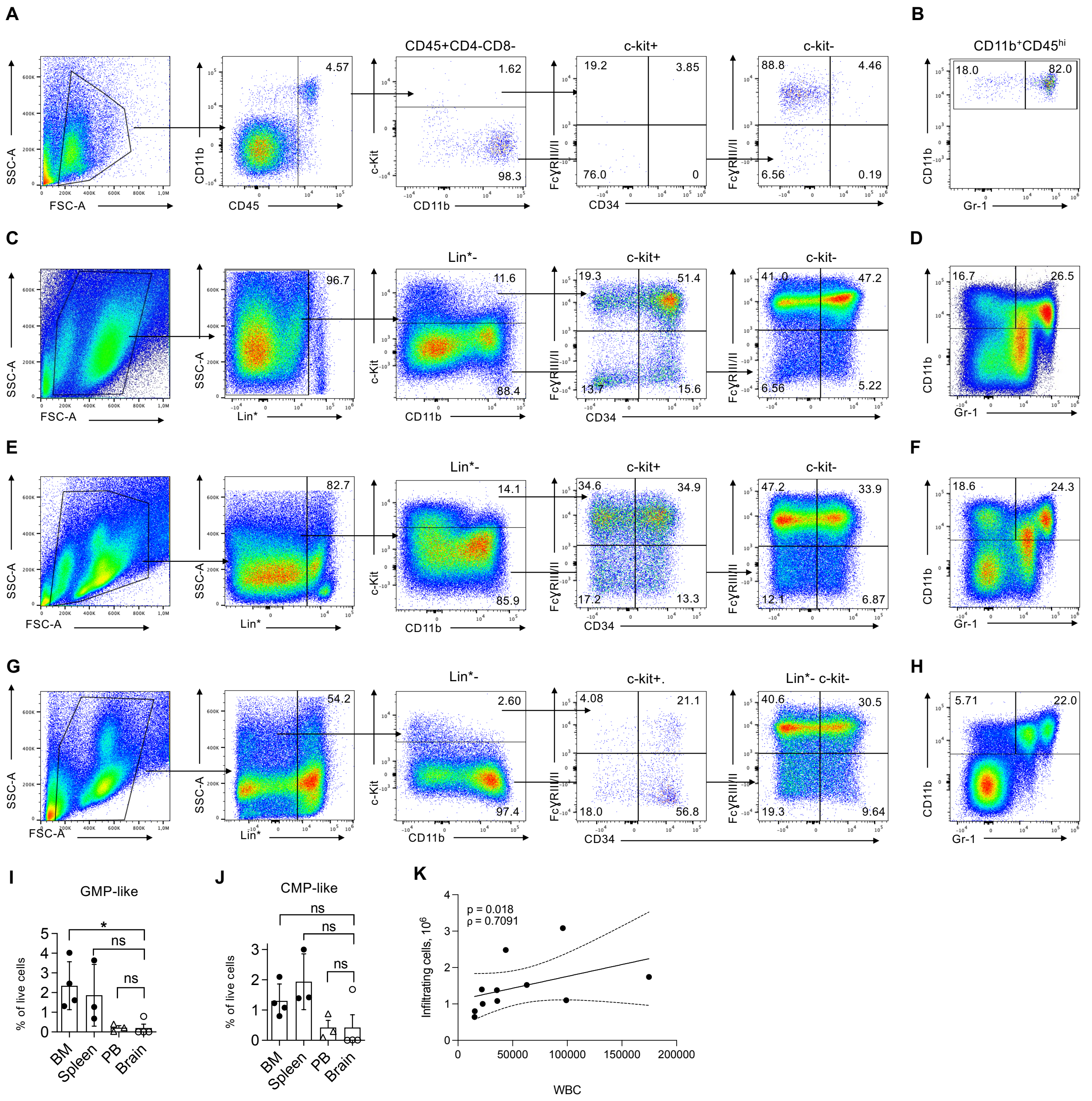

Figure S1: Characterization of myeloid populations in *WT* and *NPMc*$^{+}$*/Flt3-ITD* mice

(A) Gating strategy and representative flow cytometry plots for characterization of brain-infiltrating leukocytes in *NPM1c*$^{+}$*/Flt3-ITD* mice. Sequential gating on CD45^{+} cells following the exclusion of CD4^{+} and CD8^{+} lymphocytes and analysis of CD11b, CD34, c-Kit, and FcγRIII/II (CD16/32) expression is shown. Numbers indicate percentages within the parent gate.

(B) Representative flow cytometry plots showing Mac-1 (CD11b) and Gr-1 expression in CD45^{hi} leukocytes isolated from brains of *NPM1c*$^{+}$*/Flt3-ITD* mice.

(C,E,G) Representative flow cytometry plots showing gating strategy and the expression of myeloid progenitor markers CD34, c-Kit, and FcγRIII/II within Lin*$^{-}$ cells in bone marrow (BM) (C), spleen (E) and peripheral blood (G) of *NPM1c*$^{+}$*/Flt3-ITD* mice.

(D,F,H) Representative flow cytometry plots showing Mac-1 and Gr-1 expression in BM (D), spleen (F) and peripheral blood (H) of *NPM1c*$^{+}$*/Flt3-ITD* mice.

(I) Quantification of GMP-like cells (Lin*$^{-}$c-Kit^{+}Sca-1^{-}CD34^{+}FcγRIII/II^{+}) present in BM, spleen, PB and brain, expressed as percentage of total viable cells.

(J) Quantification of CMP-like cells (Lin*$^{-}$c-Kit^{+}Sca-1^{-}CD34^{+}FcγRIII/II^{-}) in BM, spleen, PB, and brain, expressed as percentage of total viable cells.

(K) Spearman correlation analysis between the number of infiltrating leukocytes in the brain and peripheral white blood cell (WBC) counts in *NPM1c*$^{+}$*/Flt3-ITD* mice. Spearman correlation coefficient (ρ) and p value are shown.

Each dot represents one mouse; bars indicate mean ± SEM. Data are derived from three independent experiments. (*$p < 0.05$; ns = not significant, Mann–Whitney test).

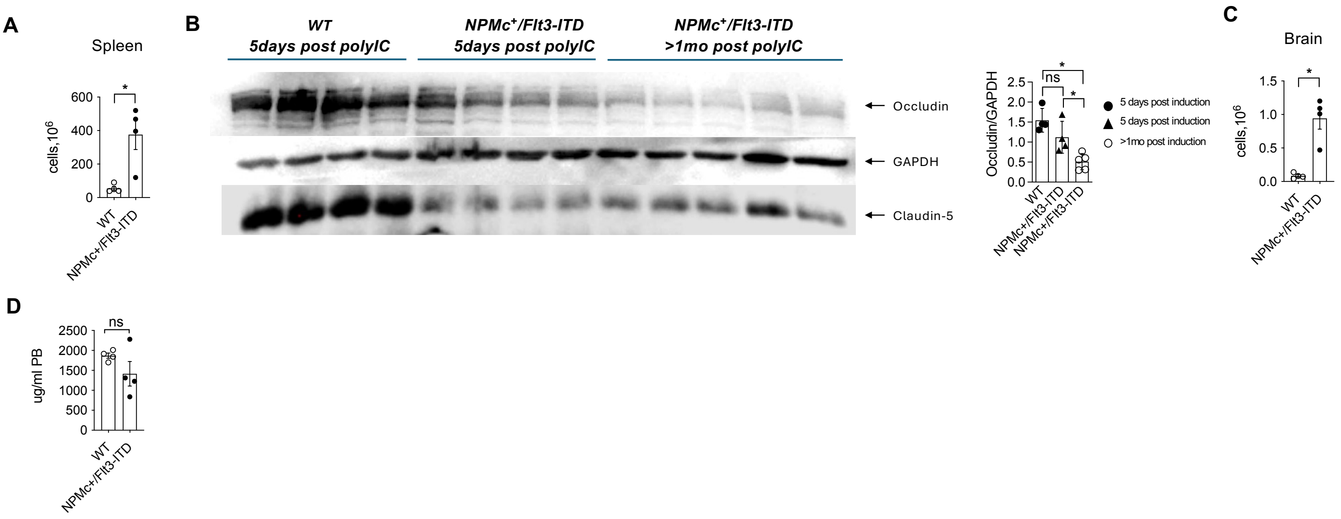

Figure S2. Blood-brain barrier alterations in *NPMc⁺/Flt3-ITD* mice

(A) Total leukocyte counts in spleen tissue of *WT* mice and *NPMc⁺/Flt3-ITD* mice 5 days after Poly(I:C) induction.

(B) Western blot analysis of occludin expression in brain lysates from *WT,* N*PMc⁺/Flt3-ITD* mice 5 days and >1 mo following Npm1c induction. GAPDH used as a loading control. Graph to the right shows quantification of occludin expression relative to GAPDH.

(C) Total leukocyte counts in brains of *WT* and *NPMc⁺/Flt3-ITD* mice 5 days after Npm1c induction.

(D) Concentration of EB (μg/mL) measured in peripheral blood (PB) of *WT* and *NPMc⁺/Flt3-ITD* mice following systemic dye injection.

Each dot represents an individual mouse; bars indicate the mean. Results are presented as means ± SEM (*$p < 0.05$; ns = not significant, Mann–Whitney test).

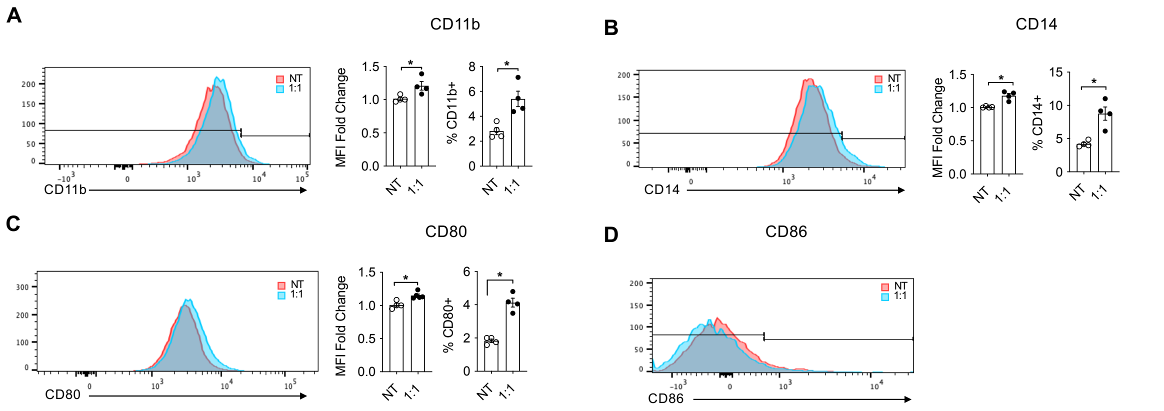

Figure S3. OCI-AML3–conditioned medium modulates myeloid and activation marker expression in HMC3 cells.

(A–D) Flow cytometry analysis of myeloid and activation marker expression in HMC3 cells cultured under non-treated (NT) conditions or exposed to OCI-AML3-conditioned medium (1:1 dilution in DMEM supplemented with 10% FBS). Overlay histograms illustrate expression of CD11b (A), CD14 (B), CD80 (C), and CD86 (D) in HMC3 cells treated with OCI-AML3-conditioned medium (light blue) compared with NT controls (red). For CD11b, CD14, and CD80, graphs report mean fluorescence intensity (MFI) fold change relative to NT controls and the percentage of positive cells. For CD86, only overlay histograms are shown, as no difference in expression was detected following treatment with OCI-AML3-conditioned medium. (*$p < 0.05$; Mann–Whitney test).

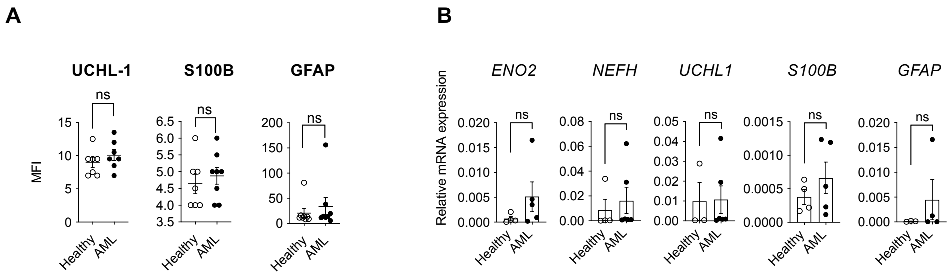

Figure S4: Expression of neuronal injury markers in peripheral blood mononuclear cells from AML patients

(A) Levels of neuronal markers in plasma of AML patients and healthy controls.

(B) Relative mRNA expression of neuronal markers (*ENO2, NEFH, UCHL1, S100B, GFAP*) in peripheral blood mononuclear cells of AML patients and healthy controls. Each dot represents an individual patient; bars indicate the mean. Results are presented as mean ± SEM of three independent experiments (ns = not significant, Mann–Whitney test).
