# Supplementary material for: Neuroinflammatory changes in acute myeloid leukemia: Evidence for blood–brain barrier disruption and glial activation
Source: Hemasphere. 2026 Mar 30;10(4):e70341. doi: 10.1002/hem3.70341 (PMC13103871; doi:10.1002/hem3.70341)
Supplement: Supplementary file 2 — Supplemental_Tables_Rev2. [file HEM3-10-e70341-s002.docx]

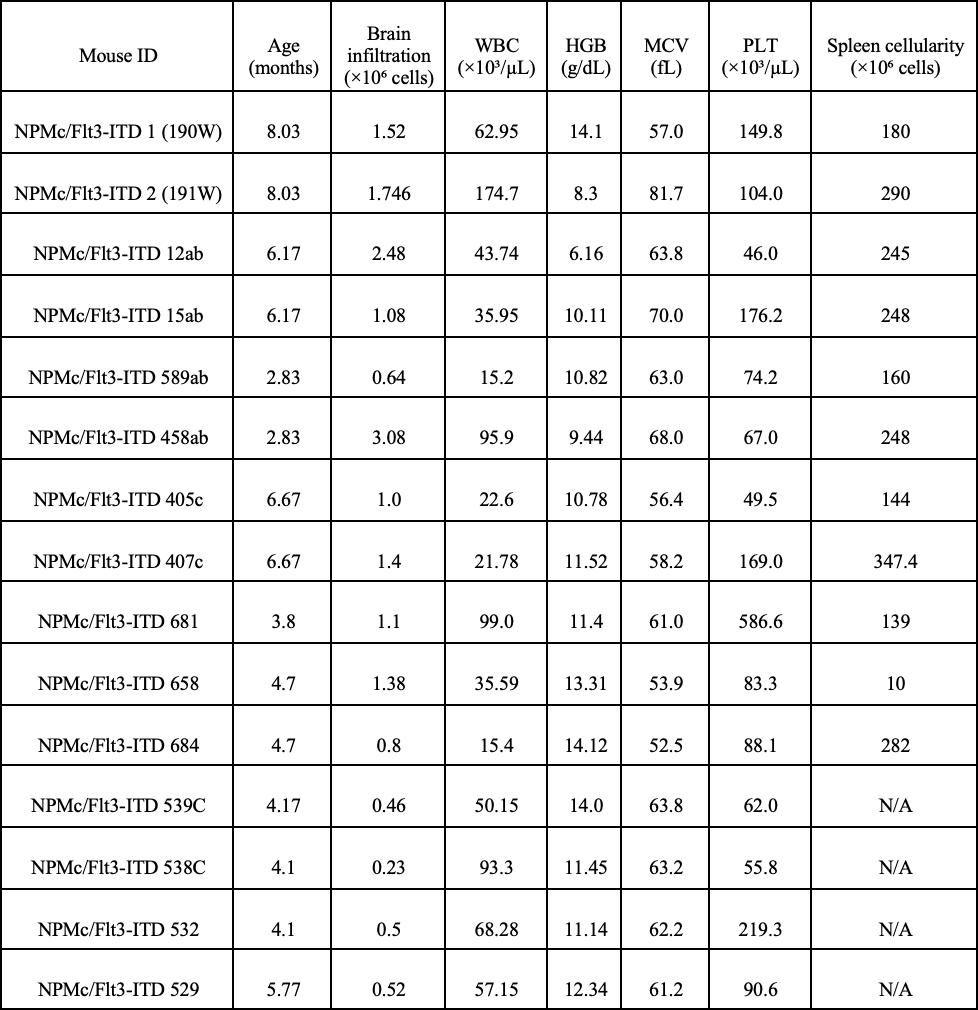


# Table 1: Individual hematologic and tissue infiltration parameters of mice included in the study

Table shows individual mouse identifiers (ID), age (in months), total leukocyte counts isolated from brain tissue (x106), blood count parameters obtained by Hemavet analysis including white blood cell (WBC, cells/µl) count, hemoglobin concentration (Hgb, g/dL), mean corpuscular volume (MCV, fL), and platelet (PLT, cells/µl ) count, as well as total spleen cellularity (x106).


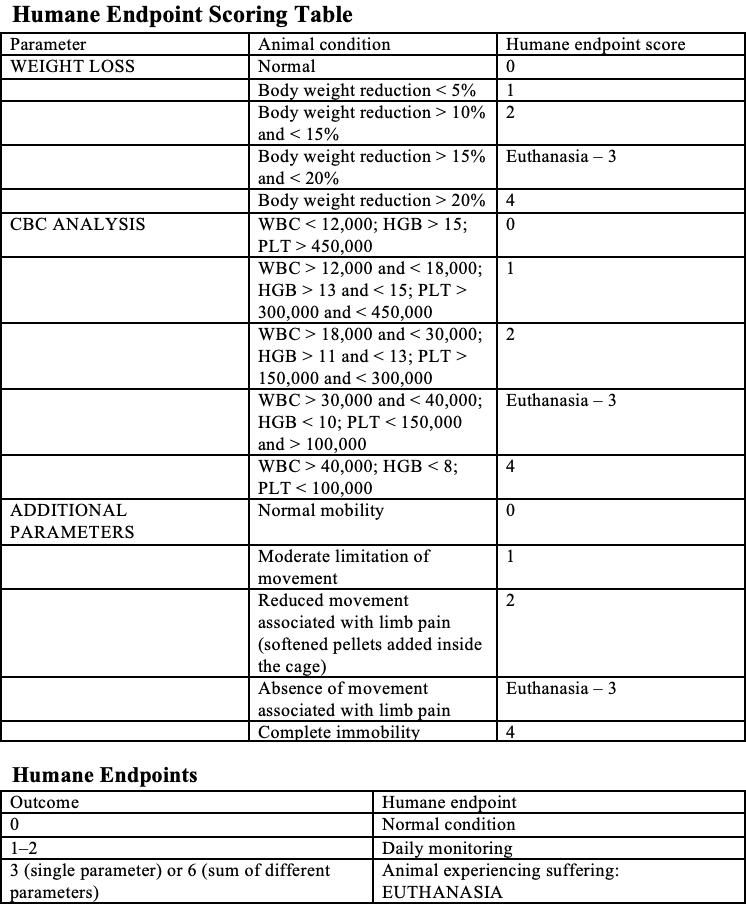


# Table 2. Humane endpoint scoring system for experimental animals.

The table summarizes the criteria used to assess animal welfare during the study, including body weight loss, complete blood count (CBC) parameters, and additional clinical signs related to mobility and pain. Each parameter is assigned a numerical score reflecting the severity of the condition. Defined score thresholds indicate the need for increased monitoring or humane euthanasia, in accordance with established animal welfare guidelines.


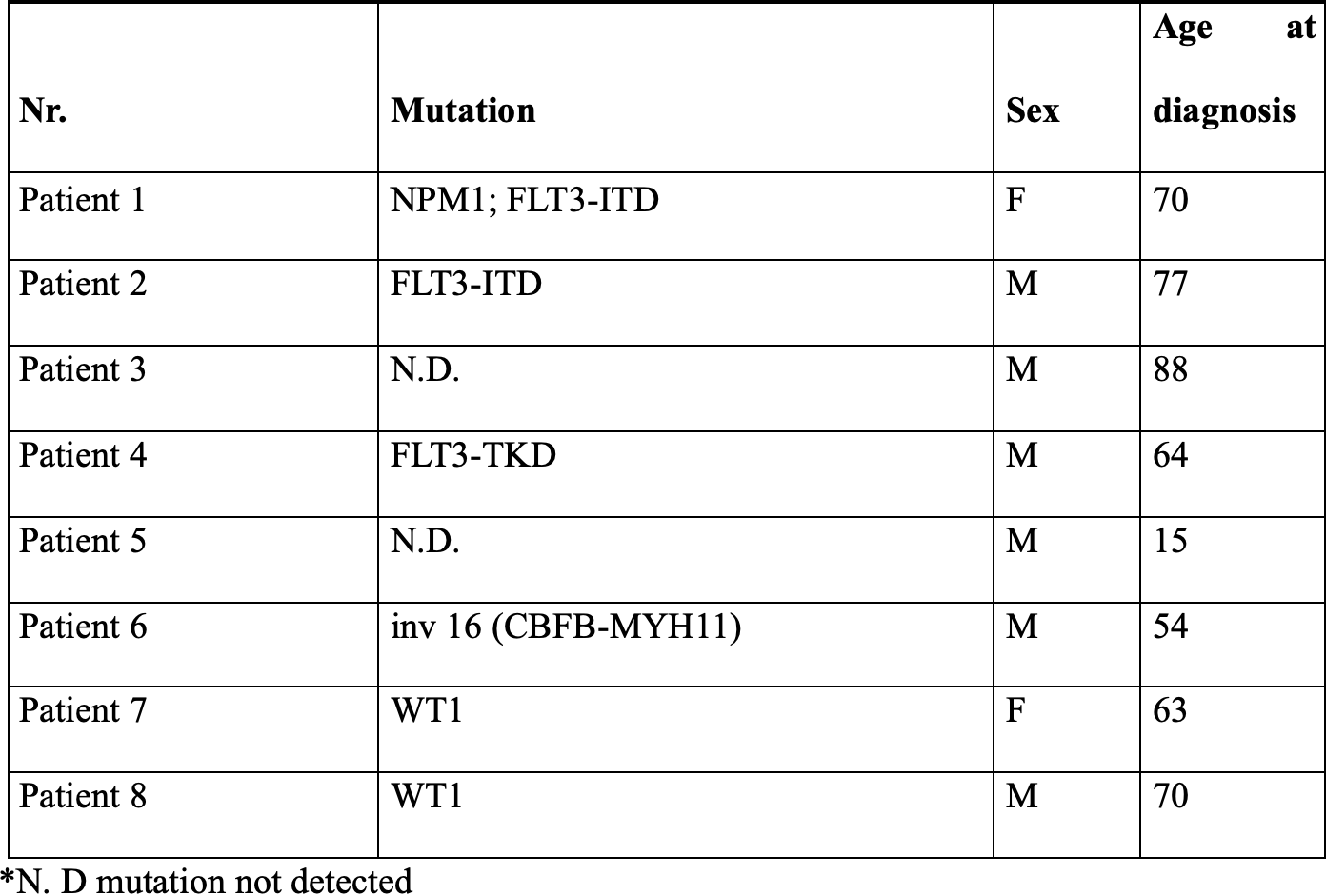


# Table 3: Clinical Characteristics of AML Cohort: Age, Sex, and Mutational Status.

Table summarizes the clinical and molecular data of patients diagnosed with acute myeloid leukemia (AML). For each patient, age, sex, and identified genetic mutations are reported.
